# Exploring In Vitro the Combination of *Cistus* × *incanus* L. and *Castanea sativa* Mill. Extracts as Food Supplement Ingredients against *H. pylori* Infection

**DOI:** 10.3390/foods13010040

**Published:** 2023-12-21

**Authors:** Giulia Martinelli, Marco Fumagalli, Carola Pozzoli, Giovanna Nicotra, Silvia Francesca Vicentini, Nicole Maranta, Enrico Sangiovanni, Mario Dell’Agli, Stefano Piazza

**Affiliations:** 1 Department of Pharmacological and Biomolecular Sciences “Rodolfo Paoletti”, University of Milan, 20133 Milan, Italy; giulia.martinelli@unimi.it (G.M.); marco.fumagalli3@unimi.it (M.F.); carola.pozzoli@unimi.it (C.P.); nicole.maranta@unimi.it (N.M.); enrico.sangiovanni@unimi.it (E.S.); stefano.piazza@unimi.it (S.P.); 2 Estratti Piante Officinali EPO s.r.l., 20141 Milan, Italy; gnicotra@eposrl.com (G.N.); svicentini@eposrl.com (S.F.V.)

**Keywords:** *Cistus*, *Cistus* × *incanus*, gastritis, *Helicobacter pylori*, inflammation

## Abstract

*Cistus* spp. have been traditionally used for inflammatory and infectious disorders, including gastrointestinal ailments, in the Mediterranean area. Among them, *Cistus* × *incanus* L. is one of the most frequently cited species in the literature for a variety of biological activities which include inflammatory diseases. *Cistus* spp. aerial parts are rich in polyphenols such as condensed and hydrolysable tannins, procyanidins, and flavonoids, which show gastroprotective activities. The purpose of the present study is to investigate the biological activities of a hydroalcoholic extract from *Cistus* × *incanus* L. aerial parts in gastric epithelial cells (GES-1) infected with *H. pylori*. The extracts inhibited IL-8 and NF-κB induced by *H. pylori* and showed antibacterial activity after simulated digestion. Since our previous paper reported interesting results on the ability of *Castanea sativa* Mill. leaf extract to decrease inflammatory conditions in *H. pylori*-infected gastric cells, the combination of *Castanea sativa* and *Cistus* × *incanus* extracts was also investigated, showing strong anti-inflammatory activity and inhibition of bacterial adhesion. This association of botanicals is proposed herein as a novel food supplement capable of counteracting gastric inflammatory conditions.

## 1. Introduction

The genus *Cistus* is traditionally used for inflammatory and infective disorders, occurring in skin, respiratory, and gastrointestinal districts, especially in the Mediterranean area [1]. *Cistus* × *incanus* L. is one of the species frequently cited in the literature for a variety of biological activities mostly demonstrated using polar extracts. The phytochemical profile of the aerial parts has been investigated by several authors, reporting that extraction with polar solvents allows the retention of a large amount of polyphenols, such as flavonols, flavan-3-ols, and tannins; the latter have been characterized as both proanthocyanidins, such as oligomeric procyanidins and prodelphinidins, and ellagitannins, such as punicalagins and cornusin B [2,3,4].

Polyphenols are considered the main compounds responsible for the antibacterial, antiviral, anti-inflammatory, and antioxidant properties ascribed to *Cistus* spp. in pre-clinical studies [2,3,4,5,6,7,8]. A key role in the biological activity might be due to the presence of condensed tannins, although few papers clearly attributed the bioactivity to specific polyphenolic fractions or individual compounds [9,10]. Condensed tannins from *Cistus* × *incanus* L., including peculiar galloylated trimers and oligomeric proanthocyanidins, are COX-2 inhibitors at low μM levels and show an anti-inflammatory effect in an in vivo model of 12-O-tetradecanoylphorbol acetate (TPA)-induced ear edema (500 mg/ear) [4]. Proanthocyanidins were also considered the main fraction responsible for the reduction of symptoms related to upper-respiratory-tract infections, according to several clinical trials [7,11]. However, at the present state of the art, the standardization of the extracts from *Cistus* spp. is limited by fragmentary knowledge of the individual compounds responsible for the efficacy of the plant extracts. 

Despite the traditional use, the biological activity of *Cistus* spp. at the gastrointestinal level has been poorly investigated. However, dietary and botanical sources of polyphenols, including tannins, are receiving growing attention for their potential role in gut inflammatory conditions [12,13]. Plant extracts rich in polyphenols were investigated for direct anti-adhesive and antibacterial properties against *Helicobacter pylori* (*H. pylori*) [14,15], a pathogen well known as the main etiologic factor of gastritis (for a deeper reading on the topic, see [16,17]). 

Our group previously characterized the early inflammatory response of non-tumoral gastric cells (GES-1) to cag+ *H. pylori* infection [18]. By the same model, we suggested that extracts from *Castanea sativa* Mill. fruit and leaf, characterized by the presence of proanthocyanidins and ellagitannins, respectively, might counteract the gastric inflammation related to *H. pylori* infection [19,20]. The proposed mechanism involves the inhibition of the NF-κB pathway and the consequent release of IL-8, which are pro-inflammatory mediators with a recognized role in the pathogenesis of gastritis [21,22]. 

To the best of our knowledge, few articles report the gastroprotective effects of *Cistus* (i.e., *incanus* or *laurifolius*) polar extracts (250–500 mg/Kg, p.o.) in animal models of chemically induced peptic ulcers [6,23]. In addition, extracts from *Cistus laurifolius* L. flowers (<250 μg/mL) showed antibacterial activity against different *H. pylori* strains [24], as well as flavonoids from *C. laurifolius* leaves (<65 μg/mL) [25], but the data are still limited, demanding further pharmacological investigations.

The present study evaluates the bioactivity of hydroalcoholic extract from *Cistus* × *incanus* L. aerial part in gastric epithelial cells (GES-1) infected with the aggressive cag+ *H. pylori* strain 26695. Hydroalcoholic extracts were chosen for their ability to retain a large number of active polyphenols such as flavonoids and tannins, according to our previous studies [19,20].

The effect on bacterial adhesion to epithelial cells and pro-inflammatory markers was investigated, and the effect correlated with the phytochemical characterization of the extract. Moreover, this study includes further investigation regarding the combination of *Cistus* × *incanus* L. aerial part and *Castanea sativa* Mill. leaf for the potential development of a novel nutraceutical product.

## 2. Materials and Methods

### 2.1. Reagents

GES-1 cells from human gastric epithelium were kindly donated by Dr. Dawit Kidane-Mulat, Howard University, College of Medicine, Washington, DC, USA. Cell culture reagents (RPMI 1640 medium, trypsin-EDTA, antibiotics, and amino acids) were provided by Gibco (Life Technologies Italia, Monza, Italy). Fetal bovine serum (FBS), and disposable material were provided by Euroclone (Euroclone S.p.A., Pero-Milan, Italy). *Helicobacter pylori* (strain 26695, cag+) was purchased from ATCC (ATCC^®^ 700392^TM^, Manassas, VA, USA). All reagents for bacterial growth and storage, including medium Mueller–Hinton Broth, Brucella Broth, and glycerol, and the gas pack to obtain the right atmosphere condition in a jar, were purchased from BD (BD, Franklin Lakes, NJ, USA). The agar was obtained from Merck Life Science (Merck Life Science, Milan, Italy), and defibrinated sheep blood was obtained from Thermo Fischer Scientific (Oxoid^TM^ Hampshire, Basingstoke, UK). The reagents for cell viability 3-(4,5-dimethylthiazol-2-yl)-2,5-diphenyltetrazolium bromide (MTT), urea, Folin–Ciocâlteu’s reagent, vanillin, AlCl_3_, natural product (2-Aminoethyl diphenylborinate), and phenol red were provided by Merck Life Science (Merck Life Science, Milan, Italy), while crystal violet was purchased from Thermo Fischer Scientific (Thermo Fischer Scientific, Basingstoke, UK). Acetonitrile, n-butanol, ethyl acetate, methanol, ethanol, formic acid, and hydrochloric acid were obtained from Merck Life Science (Merck Life Science, Milan, Italy). Carboxyfluorescein succinimidyl ester (CFSE) 5 mM (CellTrace^TM^, Cell Proliferation kits) was obtained from Invitrogen (Thermo Fisher Scientific, Waltham, MA, USA). Britelite^TM^ Plus reagent was obtained from Perkin Elmer (Perkin Elmer, Milan, Italy). Punicalagin (certified purity, >95%), punicalin (certified purity, >95%), procyanidin B1, catechin, epicatechin gallate, epigallocatechin, epigallocatechin gallate, quercetin-3-O-glucoside, and kaempferol-3-O-glucoside were obtained from Phytolab GmbH & Co. KG (Vestenbergsgreuth, Germany), while ellagic acid and chlorogenic acid were obtained from Merck. Epigallocatechin-gallate (EGCG) and procyanidin A2 were obtained from Phytolab GmbH & Co. KG (Vestenbergsgreuth, Germany) and were used for biological experiments. All the other reagents for the biological assays were HPLC-grade. All chromatographic solvents were HPLC-grade or LC–MS-grade for MS experiments. 

### 2.2. Plant Extract Preparation and In Vitro Simulated Gastric Digestion

Powder extracts from *Cistus* × *incanus* L. (CI) and *Castanea sativa* Mill. (CS) were kindly provided by EPO S.r.l. (Milan, Italy). The extracts were obtained by hydroalcoholic extraction (80:20 or 75:25 water:ethanol, respectively) of the aerial parts and leaves, respectively. Aliquots at 25 mg/mL (50:50, water:DMSO) were stored at −20 °C until biological experiments. *Cistus* × *incanus* L. extract was investigated before and after simulated gastric digestion, which was performed according to [19]. The extracts (100 mg) were subjected to 5 min incubation at 37 °C with saliva (6 mL) and for 2 h with gastric juice (12 mL). The samples were incubated at 37 °C under shaking conditions. The suspension was centrifuged (5 min, 3000× *g*) and the supernatant freeze-dried. The resulting powder (digested CI, called CI Dig.) was used for the experiments.

### 2.3. LC–MS/MS and TLC Analysis

*Cistus* × *incanus* L. extract and *Castanea sativa* Mill. leaf extracts were analyzed by UPLC–MS/MS analysis using Exion LCTM AC System (AB Sciex, Foster City, CA, USA) coupled to a Triple Quad^TM^ 3500 system (AB Sciex, Foster City, CA, USA) with an ESI (−) source. The compounds were separated with a Synergi 4 μm Hydro-RP 80 A LC Column 150 × 4.6 mm (Phenomenex, Torrance, CA, USA) using a mobile phase consisting of water with 0.1% formic acid (A) and methanol (B) set as follows (Table 1).

The injection volume was 5 μL for each sample. Mass-spectrometric detection was performed in negative ionization (ESI) mode, and the parameters were set as follows: curtain gas at 40 psi, ionization voltage at −4500 V, source temperature at 500 °C, and nebulization gas 1 and nebulization gas 2 at 50 psi. The optimized compound-dependent MS/MS parameters (entrance potential, declustering potential, collision cell exit potential, and collision energy) were obtained by a separate injection of the analytes (multiple-reaction monitoring, MRM). The LC–MS/MS system was under the control of AB Sciex Analyst (version 1.7) software.

TLC analysis of the polyphenolic profile was performed according to the textbook from Wagner and Bladt [26]. In brief, 500 μg of extract (10 μL of 50 mg/mL methanol stock solution) was seeded on the silica layer 60 F254 (10 × 20 cm, Merck, Darmstadt, Germany). The mobile phase was prepared with ethyl acetate–acetic acid–formic acid–water (100:11:11:26), while derivatization at the end of the run was conducted by spraying Natural Product reagent (2-aminoethyl diphenylborinate, 1% methanol solution) on the layer. The phenolic profile was revealed and acquired under a UV 366 nm lamp (software VisionCats 2.0, CAMAG, Muttenz, Switzerland).

### 2.4. Total Phenol Content

Total Polyphenol Content (TPC) was determined using Folin–Ciocâlteu’s method [27]. Briefly, freeze-dried leaf extracts (1 mg) were solubilized in 1 mL of water. Then, 300 μL of diluted extract was mixed with diluted Folin–Ciocâlteu’s reagent (1:10) and 1.2 mL of 7.5% (*w*/*v*) sodium carbonate, reaching a final volume of 3 mL. The absorbance of the sample (at 765 nm) was compared with the absorbance of gallic acid (GA), used at different concentrations to obtain a standard calibration curve (0–30 μg/mL) (Victor^TM^ X3, Perkin Elmer, Walthman, MA, USA). The total amount of polyphenols was measured as GA equivalents (GA eq.) then presented as weight of dry extract GA eq./weight of GA (%).

### 2.5. Total Flavonoids Content

Aluminum chloride (AlCl_3_) is widely used for general qualitative and quantitative analysis of flavonoids, with specific reference to flavonols [28]. In brief, 20 μL of sample extract, or standard reference flavonol (quercetin), diluted in methanol (10 mg/mL and 1 mg/mL, respectively), was combined with 20 μL of AlCl_3_ (10% *w*/*v*), 20 μL of CH_3_COOK (1 M), 560 μL of water, and 380 μL of methanol, with a final volume of 1 mL. The blank of each sample was also prepared by excluding AlCl_3_ salt from the mixture. The development of a bright yellow-orange color comparable to quercetin indicates the presence of flavonoids. For quantitative purpose, the reference calibration curve was made by serial dilution of quercetin (0–20 μg/mL) before the addition of salts. The absorbance at 430 nm was read by a photometer (JASCO V630 International Co., Ltd., Tokyo, Japan) and results were then expressed as average weight (w.) of quercetin equivalents (eq.) on w. of extract (%) ± SEM.

### 2.6. Total Flavan-3-ols and Proanthocyanidins Content

The vanillin assay is widely used to determine and quantify the presence of polyphenols bearing a catechol group in plant extracts, namely flavan-3-ols and their derivatives of condensation (proanthocyanidins) [29]. Thus, positive results reflect the presence of flavan-3-ol units, without indicating the nature and degree of polymerization. On the contrary, the hydrolysis with n-butanol/HCl 5% (n-butanol assay) is widely used to determine the presence of oligomeric proanthocyanidins [30]. For this reason, vanillin assay was compared with n-butanol assay to address and estimate the presence of monomeric or polymeric flavan-3-ols. 

In brief, a freshly prepared solution of 1% vanillin is added to 8% HCl diluted in methanol, with a ratio of 1:1 (working solution). The working solution is kept warmed at 30 °C, while samples and standard reference compound (catechin) are diluted in a final volume of 500 μL of methanol. Catechin is serially diluted to obtain a reference calibration curve (0–200 μg/mL). Then, 2.5 mL of working solution is added to the samples and incubated at 30 °C (time: 20 min). After that, the absorbance at 500 nm (pink-red) is immediately read (JASCO V630 International Co., Ltd., Tokyo, Japan). The results are expressed as average w. of catechin eq. on w. of extract (%) ± SEM.

N-butanol assay was conducted by adding 10 mg of powder extract to 3 mL of butanol/HCl 5% solution. Then, the samples were warmed in a water bath at 90 °C for 2 h. The development of a wine-red color is related to the presence of proanthocyanidins. The quantification was conducted by adaptation from the method reported in *Crataegi fructus* monograph, European Pharmacopeia 11. An extract from chestnut peel (1 mg), previously characterized for the high content oligomeric proanthocyanidins [20], was included for a better comparison. The absorbance of the sample at 500 nm was compared with a calibration curve made by serial dilution of cyanidin chloride (0–100 μg/mL). The results are then expressed as average w. of cyanidin eq. on w. of extract (%) ± SEM.

### 2.7. Bacterial Culture

The 26695 *H. pylori* strain (cag+) was cultured as previously mentioned [19]. Briefly, the bacterial was grown on Petri dishes with Mueller–Hinton Broth medium, 5% agar, and 25% sheep blood in a microaerophilic atmosphere (5% O_2_, 10% CO_2_, and 85% N_2_) at 37 °C and 100% humidity for 72 h. Then, the bacterium was collected and counted by optical density (at 600 nm), considering that an OD value of 5 corresponds to 2 × 10^8^ bacteria. The OD value was adjusted to test the antibacterial activity and perform the co-culture with GES-1 cells.

### 2.8. Cell Culture and Treatment

Human gastric epithelial (GES-1) cells were cultivated in RPMI 1640 medium, supplemented with penicillin 100 units/mL, streptomycin 100 mg/mL, L-glutamine 2 mM, and 10% heat-inactivated FBS. Cells were incubated at 37 °C, 5% CO_2_, in a humidified atmosphere. Cells were subcultured every 48–72 h.

Adherent GES-1 cells were infected with a suspension of *H. pylori* with a bacteria-to-cell ratio of 50:1 for 1 h or 6 h. Extracts were added at different concentrations (0–200 μg/mL). The infection treatments were carried out with a serum- and antibiotic-free medium. To allow cell synchronization, serum starvation was performed the day before treatment, using 0.5% FBS medium, supplemented with 1% L-glutamine and 1% penicillin/streptomycin. Infected cells were maintained in an aerobic atmosphere, with 5% CO_2_ throughout the treatment period. EGCG (50 μM) and procyanidin A2 (500 μM) were used as reference inhibitors of inflammatory markers or bacterial adhesion, respectively [15,19,31].

### 2.9. Cytotoxicity Assay

Cell morphology was evaluated by light microscope inspection before and after each treatment to check integrity. The toxicity of the plant extract, in the range of 0–200 μg/mL, was excluded using the MTT method [32]. Briefly, GES-1 cells were seeded in a 24-well plate (3 × 10^4^ cells/well); the viability was measured at the end of the treatment (6 h) by adding 200 μL of diluted MTT solution (0.1 mg/mL, PBS 1×) to each well for 45 min. Then, the MTT solution was removed, and the purple formazan salt included in the cells was dissolved by isopropanol/dimethyl sulfoxide (DMSO) buffer (90:10 *v*/*v*). The viability was directly correlated to the absorbance, read at 595 nm (Victor^TM^ X3, Perkin Elmer, Walthman, MA, USA) and corrected by subtracting the blank value (solvent alone).

### 2.10. Measurement of IL-8 Release

Cells were seeded at 3 × 10^4^ cells/well (24-well plate) for 48 h before treatment (6 h) with *H. pylori* and the extracts. IL-8 was quantified in 100 μL of cell medium by ELISA assay (Human IL-8 ABTS Standard ELISA Development Kit, Peprotech, UK), according to manufacturer’s instructions [19]. The absorbance was read at 405 nm and compared to the absorbance of the standard IL-8 (0–1000 pg/mL). The mean ± SEM of each sample was compared to the stimulated control (*H. pylori* infection), as a relative percentage. For this reason, the stimulated control was arbitrarily assigned to the value of 100%.

### 2.11. NF-κB Activation

Cells were cultivated on coverslips placed in 24-well plates (3 × 10^4^/well) for 24 h. The translocation of p65 (NF-κB) into cell nuclei, reflecting the activation of the NF-κB pathway, was evaluated using immunofluorescence. Three independent experiments were conducted. *H. pylori* was stained with 5 mM CFSE (2 μL of CFSE: 5 × 10^8^ bacteria) and incubated for 20 min at 37 °C. The bacterial suspension was supplemented with FBS, washed three times (PBS 1×), and centrifuged at 3150× *g* for 5 min to remove CFSE not bound to the bacterium. Then, the cells were infected, as previously reported, and treated with the extracts at the concentration of 50, 100, and 200 μg/mL for 1 h. After 1 h, the coverslips were washed (PBS 1×) and fixed with 4% formaldehyde solution for 15 min at r.t. Then, a blocking solution (5% BSA) was added to the well and incubated at room temperature for 1 h. Finally, cells were stained with the primary antibody anti-p65 (D14E12, XP^®^ Rabbit mAb #8242, Cell Signaling Technology, Danvers, MA, USA) diluted 1:400 *v*/*v* and placed overnight at 4 °C. The day after, the coverslips were washed three times (PBS 1×) and incubated for 2 h with the secondary antibody (Alexa Fluor 647-conjugated anti-rabbit IgG, Cell Signaling Technology, Danvers, MA, USA), diluted 1:1000 *v*/*v*. To stain the cytoskeleton, a drop of ActinRed^TM^ 555 ReadyProbes^TM^ reagent (Invitrogen, Thermo Fisher Scientific, Waltham, MA, USA), diluted 1:5 in PBS 1×, was added 30 min before the end of incubation. Then, coverslips were washed again with PBS 1× and mounted on slides with a drop of ProLong Gold Antifade Reagent with 4′,6-diamidino-2-phenylindole (DAPI) (Cell Signaling Technology, Danvers, MA, USA) to stain cell nuclei. The images were obtained using a confocal laser scanning microscope (LSM 900, Zeiss, Oberkochen, Germany).

### 2.12. Bacterial Adhesion to Cells

Bacterial adhesion was measured by a cytofluorimetric method according to Messing and colleagues, with minor modifications [31]. Briefly, GES-1 cells were seeded in 12-well plates for 48 h (6 × 10^4^ cells); then, cells were infected with CSFE-stained *H. pylori* (2 μL of CFSE for 10^8^ bacteria) for 1 h. At least three independent treatments were carried out, treating cells with plant extracts for 1 h before or simultaneously to bacterial infection, in the range of 50–200 μg/mL. The anti-adhesive natural compound procyanidin A2 (500 μM) was used as a reference inhibitor. At the end of the treatment, cells were fixed by formaldehyde (4% in PBS), centrifuged, washed (PBS 1×), and resuspended in 0.5% BSA (PBS/EDTA 2 mM) for the cytometric analysis (NovoCyte flow cytometer, ACEA Biosciences, San Diego, CA, USA).

### 2.13. Minimum Inhibitory Concentration (MIC)

The microbroth dilution method was carried out following the recommendations of the Clinical and Laboratory Standards Institute [33] to evaluate the MIC value. The extract was tested in comparison to tetracycline (0.125 μg/mL). Then, 100 μL of each sample was placed in a 96-well U-bottom plate, along with 100 μL of *H. pylori* suspension (OD = 0.1). The plate was incubated at 37 °C (5% CO_2_) under microaerophilic conditions for 72 h. The rate of bacterial growth was measured, after shaking, by optical density at 600 nm (Victor^TM^ X3, PerkinElmer, Waltham, MA, USA).

### 2.14. Urease Activity

The enzymatic activity of urease from *H. pylori* was measured by a colorimetric assay, based on the conversion of urea to ammonia (NH_3_), according to a previous article [34]. The production of NH_3_ generates an increase in pH, detectable by the pH indicator phenol red (0.001 g/L), diluted in a phosphate buffer containing urea 2% *w/v* (9.1 g/L KH_2_PO_4_, 9.5 g/L Na_2_HPO_4_). Plant extracts were aliquoted in 96-well plates in the presence or absence of adherent GES-1 cells (seeded 10^4^ cells/well for 48 h). Then, 100 μL of bacterial suspension (OD = 0.1, PBS 1×) and 100 μL of 2% urea buffer were added. NaF (1 mM), known as a chelating agent of Ni^2+^, was used as a reference inhibitor [34]. The shift of absorbance to 570 nm was measured using a spectrophotometer after 1 h (Victor^TM^ X3, PerkinElmer, Waltham, MA, USA).

### 2.15. Biofilm Formation

MBEC (Minimum Biofilm Eradication Concentration) was measured by crystal violet (CV) assay [35]. Firstly, the microbroth dilution was conducted in 96-well U-bottom plates, as mentioned for MIC calculation. At the end of the bacterial growth in the presence of plant extract (72 h, microaerophilic environment), the planktonic bacteria were removed. The adherent bacteria were washed one time with PBS 1× and fixed with 50 μL of 4% formaldehyde for 5 min. The plate was centrifuged (3000× *g*, 3 min.) before removing the residual formaldehyde. After washing again, the biofilm was dried at 60 °C in a stove for 15 min. Finally, the biofilm was stained with 50 μL of CV solution (0.1%) for 15 min; then, the excess was removed by washing 2 times with PBS 1×, and a solution of acetic acid (33% *v*/*v*) was added to solubilize the purple crystals. The absorbance at 600 nm was read (Victor^TM^ X3, PerkinElmer, Waltham, MA, USA) and compared with the control conditions (*H. pylori* samples), arbitrarily attributed to the value of 100% of biofilm formation. The blank absorbance of each sample, obtained by following the same procedure in the absence of bacteria, was subtracted from the final value to consider the possible interference of colored natural compounds.

### 2.16. Statistical Analysis

The results were expressed as the mean ± SEM of at least three independent experiments; the confidence interval (95% C.I.) related to the half-maximal inhibitory concentrations (IC_50_) was reported. The p value of biological data was elaborated using ANOVA and Holm post hoc analysis, while phytochemical measurements were assessed by an unpaired *t* test (CI vs. CI Dig.). Statistical assessment and IC_50_ calculation were conducted using GraphPad Prism 9.0 software (GraphPad Software Inc., San Diego, CA, USA). Values of *p* < 0.05 were considered statistically significant.

## 3. Results

### 3.1. Phytochemical Analysis

The bioactivity of polar extracts from *Cistus* × *incanus* L. was previously attributed to polyphenols, including flavonoids and proanthocyanidins. As the first step of this study, a hydroalcoholic extract from *Cistus* × *incanus* L. (CI) was generally characterized for the presence of polyphenols by TLC analysis and Folin–Ciocâlteu’s method; then, specific assays for the phenolic subclasses of flavonoids and tannins were carried out. All the assays were repeated after the simulation of the gastric digestion to investigate the potential stability of polyphenols in the gastric environment. 

**Figure A1:**
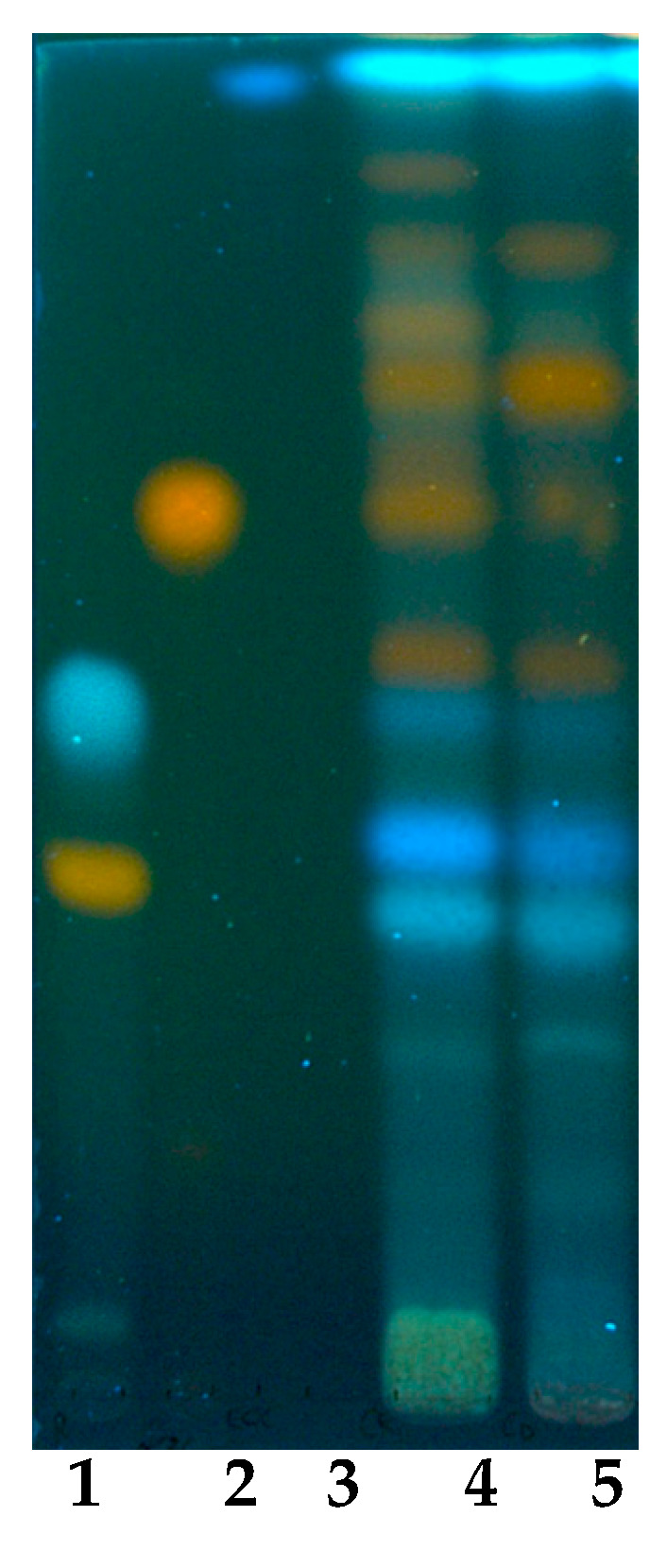
TLC analysis of polyphenols from *Cistus* × *incanus* L. (CI). 1. Chlorogenic acid (light blue) and rutin (orange) (10 μg); 2. Hyperoside (10 μg); 3. Epicatechin (10 μg); 4. CI (500 μg); 5. CI Dig. (500 μg).

The TLC analysis (Figure A1) showed a rich polyphenolic profile, characterized by flavonol-glycosides (orange stripes), such as hyperoside, and caffeoylquinic acids (light blue stripes) to a lesser extent, including chlorogenic acid. It was also difficult to exclude the presence of monomeric catechins, such as epicatechin (blue). Of note, this polyphenolic profile was barely affected by the simulated gastric digestion.

As summarized in the column CI of Table 2 (Table 2), the Folin–Ciocâlteu assay verified that polyphenols represent a relevant fraction of the extract, specifically 25.63% of GA eq. (*w/w* dry extract). Comparable quantitative results were obtained by vanillin assay (24.19% of catechin eq.), thus suggesting that flavan-3-ols and proanthocyanidins may represent the largest part of the polyphenolic fraction. Results from the AlCl_3_ assay were informative regarding flavonoids, with specific reference to flavonols: they were already revealed by TLC analysis and represented a minor but still relevant fraction of polyphenols (1.35% of quercetin eq.). With regard to proanthocyanidins, we performed a modified n-butanol/HCl assay with quantitative purpose, thus measuring 2.27% of cyanidin eq. 

To address the stability of polyphenols in quantitative terms, the gastric digestion was simulated in vitro, and the colorimetric assays were repeated on digested samples (Table 2, column CI Dig.). The simulated digestion showed a statistically significant impact on the stability of total polyphenols, which decreased from 25.63% to 15.49% of GA eq. Accordingly, the vanillin test showed a significant decrease to 12.53% of catechin eq., and proanthocyanidins were highly degraded. On the contrary, the level of flavonols was not altered.

The overall colorimetric data prompted a deeper characterization of flavonols and catechins by LC–MS: the latter were intended as possible explanation of the quantitative gap among vanillin-reactive compounds and proanthocyanidins. In line with Wittpahl and colleagues [2], the analytical assay identified the presence of flavonols, such as quercetin-3-O-glucoside 463 (301), kaempferol-3-glucoside 447 (284), and myricitrin-3-rhamnoside 463 (318): the first two compounds were quantified by analytical standards (Table 3). Moreover, the ellagitannin punicalagin was identified as a minor compound. Accordingly, the derivatives of hydrolysis punicalin and ellagic acid were measured as well. 

### 3.2. Biological Effect of Cistus × incanus L. Extracts on Markers of Inflammation in GES-1 Cells Infected by H. pylori

**Figure A2:**
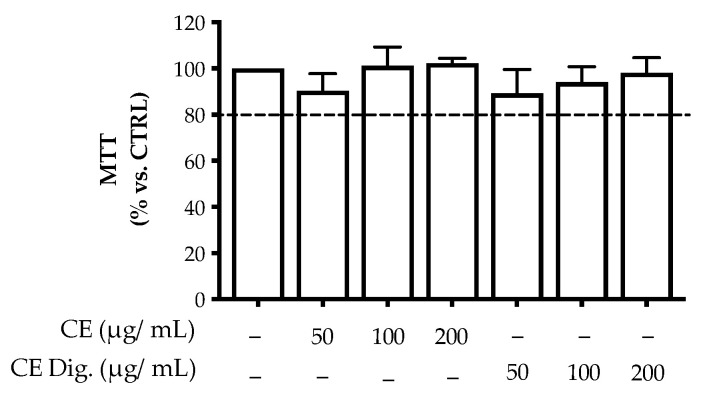
Effect of *Cistus* × *incanus* L. extract on GES-1 viability (MTT test). GES-1 cells were treated with CI or CI Dig. for 6 h. Results (*n* = 3) were expressed as a percentage (%) of viability (absorbance at 595 nm) ± SEM, relative to untreated cells. The dashed line represents the cut-off for acceptable value of viability, arbitrarily considered above 80%.

The second phase of our study was aimed at investigating the bioactivity of CI in gastritis through a model of human non-tumoral gastric epithelial cells (GES-1) infected with *H. pylori* cag+ strain 26695 (ATCC^®^) [18]. The previous analytical data suggested that the stability of the polyphenolic fraction was partially affected by the gastric environment. For this reason, the bioactivity of CI was investigated in comparison with CI Dig. The extracts reported no significant alteration of cell viability up to the concentrations of 200 μg/mL (Figure A2).

**Figure 1:**
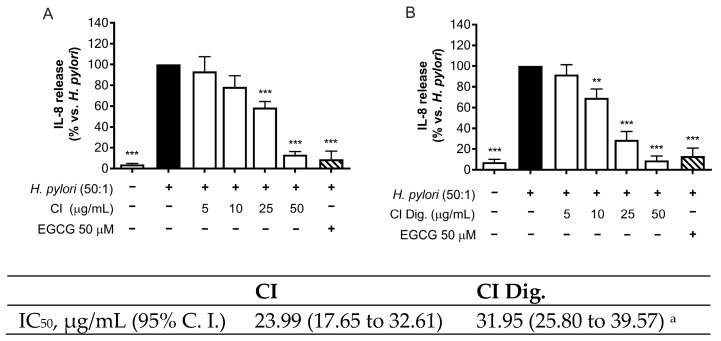
Effect of *Cistus* × *incanus* L. extracts on IL-8 release induced by *H. pylori* in GES-1 cells. GES-1 cells were infected with *H. pylori* (50:1) and treated with CI (**A**) or CI Dig. (**B**) for 6 h. EGCG (50 μM) was used as a reference inhibitor. IL-8 secretion was measured through ELISA assay. Results (*n* = 3) were expressed as a percentage (%) of the mean of IL-8 release (pg/mL) ± SEM, relative to *H. pylori* infection (black bar). ** *p* < 0.01, *** *p* < 0.001 vs. *H. pylori* infection. ^a^ *p* < 0.0001 of CI dig with respect to CI.

*H. pylori* is known to activate NF-κB and induce the release of the chemokine IL-8 in the gastric mucosa [21]. In our experiments, CI inhibited the release of IL-8 in GES-1 cells in a concentration-dependent fashion (IC_50_ = 23.99 μg/mL) (Figure 1A). The inhibitory activity was also demonstrated after simulated gastric digestion, although slightly impaired, as underlined by the comparison of the respective IC_50_ (from 23.99 to 31.95 μg/mL) (Figure 1B).

**Figure 2:**
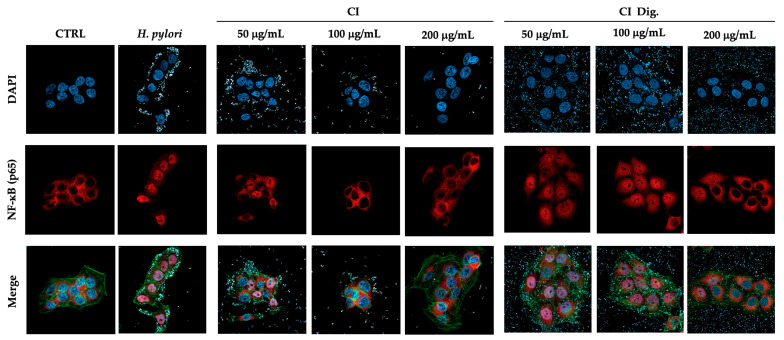
Effect of *Cistus* × *incanus* L. extracts on the nuclear translocation of NF-κB (p65) induced by *H. pylori* in GES-1 cells. GES-1 cells were infected with *H. pylori* (50:1) and treated with CI or CI Dig. for 1 h. NF-κB (subunit p65, red) translocation into nuclei (blue) was measured by immunofluorescence (63× objective, 50 μm). Merged images include β-actin (green) and bacterial staining (CFSE, white).

To address a possible mechanism of action and sustain the evidence of potential anti-inflammatory activity, CI was tested against the NF-κB pathway. Immunofluorescence assays showed that CI impaired the translocation of p65 into GES-1 cell nuclei during *H. pylori* infection at 100 and 200 μg/mL. The inhibitory activity of CI Dig. was observed starting with a higher concentration (200 μg/mL), in line with the previous experiment (Figure 2). Of note, the detachment of the bacteria from cell surface was clearly notable at the same concentrations responsible for the interference with NF-κB translocation. This observation prompted the study of the anti-adhesive and antibacterial properties of the extract, as further modes of action. 

### 3.3. Biological Effect of Cistus × incanus L. Extracts on Factors Involved in the Colonization of H. pylori

**Figure 3:**
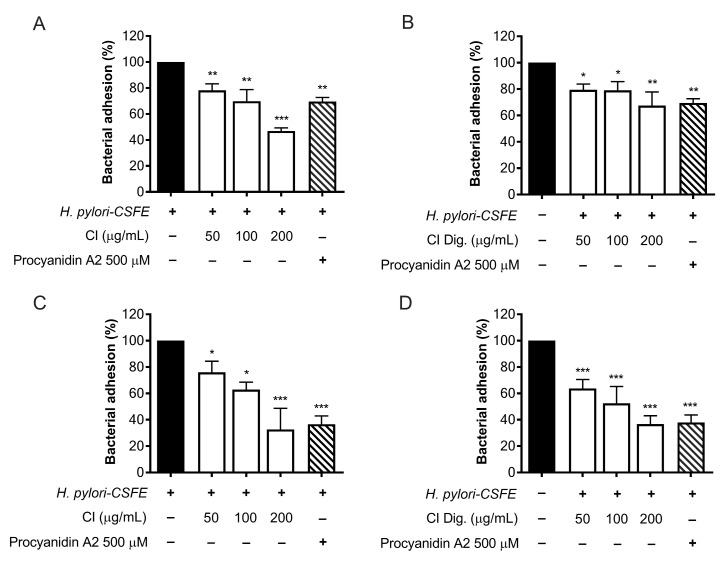
Effect of *Cistus* × *incanus* L. extracts on the adhesion of *H. pylori* to GES-1 cells. GES-1 cells were infected with *H. pylori–CSFE* (50:1) and treated with CI (**A**,**C**) or CI Dig. (**B**,**D**) for 1 h, before (**A**,**B**) or after (**C**,**D**) infection. Procyanidin A2 (PA, 500 μM) was used as a reference inhibitor. Bacterial adhesion was measured by FACS analysis. Results (*n* = 3) were expressed as a percentage (%) of the mean bacterial adhesion (MFI) ± SEM, relative to untreated *H. pylori* (black bar). * *p* < 0.05, ** *p* < 0.01, *** *p* < 0.001 vs. *H. pylori* infection. MFI, median fluorescence intensity.

The adhesion and outgrowth of *H. pylori* in the gastric mucosa are strongly related to virulence factors, including the expression of cag genes and the enzymatic activity of urease [16]. The anti-adhesive properties of CI were investigated by cytofluorimetric analysis, using fluorescent-labeled (CSFE) *H. pylori*. CI impaired the adhesion to gastric cells at 100 and 200 μg/mL; the bioactivity was observed either by treating GES-1 cells before (pre-treatment) and along with (co-treatment) the infection (Figure 3A,C), thus suggesting the involvement of actions directed to both human and bacterial cells. The simulated digestion showed a slight impact on the inhibitory activity, in the pre-treatment setting only (Figure 3B). On the contrary, a marked inhibition, comparable to the reference inhibitor procyanidin A2, was maintained at 200 μg/mL in the co-treatment setting (Figure 3D). 

**Figure 4:**
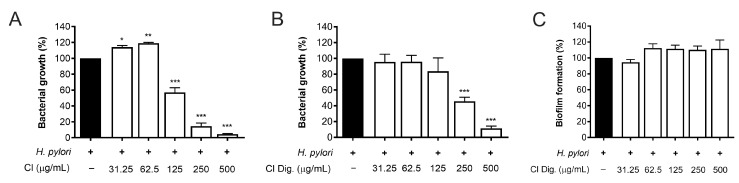
Effect of *Cistus* × *incanus* L. extracts on *H. pylori* growth and biofilm formation. Bacteria were treated with CI (**A**) or CI Dig. (**B**,**C**) for 72 h. Tetracycline (MIC = 0.125 μg/mL) was used as a reference antibiotic in each experiment. MIC values (**A**,**B**) were obtained by microbroth dilution, while biofilm formation was revealed by crystal violet assay. Results (*n* = 3) were expressed as a percentage (%) of the mean bacterial growth (absorbance at 600 nm) ± SEM, relative to untreated *H. pylori* (black bar). * *p* < 0.05, ** *p* < 0.01, *** *p* < 0.001 vs. *H. pylori* infection.

The experiments on bacterial adhesion paralleled the previous microscopy results (Figure 2), thus underlining the direct effect of the extract on *H. pylori*. Consequently, this property was investigated by microbroth dilution, thus obtaining MIC values of 125 and 250 μg/mL for CI and CI Dig., respectively (Figure 4A,B). On the contrary, the CI Dig. was unable to counteract the formation of bacterial biofilm, addressed by crystal violet assay, till reaching a concentration of 500 μg/mL (Figure 4C).

**Figure 5:**
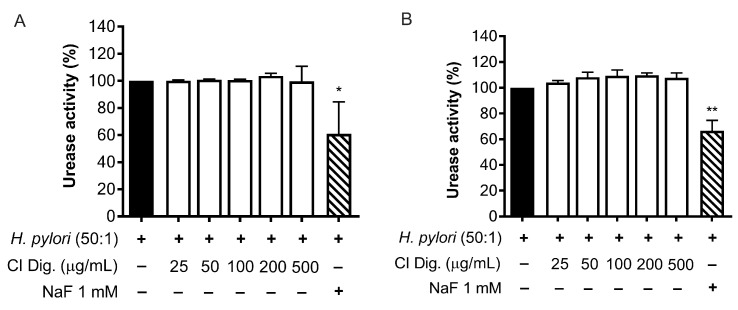
Effect of *Cistus* × *incanus* L. extract on *H. pylori* urease activity. Adherent *H. pylori* to GES-1 (**A**) or *H. pylori* suspension (**B**) were treated with CI Dig. for 1 h. NaF (1 mM) was used as a reference urease inhibitor. The enzymatic activity was assessed using a colorimetric assay based on pH indicator. Results (*n* = 3) were expressed as a percentage (%) of pH increase (absorbance at 570 nm) ± SEM, relative to untreated *H. pylori* (black bar). * *p* < 0.05, ** *p* < 0.01 vs. *H. pylori* infection.

A recognized factor for the success of the bacterial colonization is the urease activity of *H. pylori*. We tested the effect of CI Dig. on the enzyme by treating a suspension of bacteria or bacteria cultured with GES-1 cells (Figure 5A,B). In our experimental settings, the extract (25–500 μg/mL) was unable to interfere with the enzymatic conversion of urea to ammonia, in contrast to the reference inhibitor NaF (1 mM).

### 3.4. Biological Activity of a Combination of Extracts from Cistus × incanus L. and Castanea sativa Mill

The experimental data about CI showed a more potent inhibitory activity on *H. pylori* adhesion than NF-κB pathway. In fact, CI Dig. showed significant anti-adhesive effect at 50 μg/mL, while NF-κB translocation was clearly impaired only at 200 μg/mL. 

In our previous study on *Castanea sativa* Mill. leaf extract (CS) [19], we observed an opposite trend, since the extract impaired the NF-κB pathway at concentrations lower than 200 μg/mL and bacterial adhesion at concentrations higher than 100 μg/mL. 

For this reason, we wondered whether the combination of CI and CS might result in complementary effects against *H. pylori* infection.

**Figure 6:**
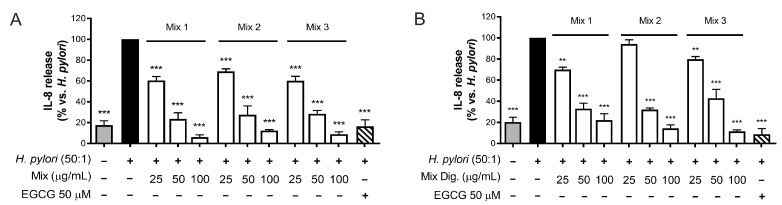
Effect of combinations (mix) of *Cistus* × *incanus* L. (CI) and *Castanea sativa* Mill. (CS) extracts on IL-8 release induced by *H. pylori* in GES-1 cells. GES-1 cells were infected with *H. pylori* (50:1) and treated with mix (**A**) and mix Dig. (**B**) for 6 h. EGCG (50 μM) was used as a reference inhibitor. IL-8 release was measured by ELISA assay. Mix 1, combination of CI/CS 75:25; Mix 2, combination of CI/CS 50:50; Mix 3, combination of CI/CS 25:75. Results (*n* = 3) were expressed as a percentage (%) of the mean of IL-8 release (pg/mL) ± SEM, relative to *H. pylori* infection (black bar). ** *p* < 0.01, *** *p* < 0.001 vs. *H. pylori* infection.

As the first step, we tested the inhibitory activity on IL-8 after mixing the two extracts by three different ratios of CI:CS (*w*/*w*): 75:25 (Mix 1), 50:50 (Mix 2), and 25:75 (Mix 3). The results shown in Figure 6 (Figure 6A,B) demonstrated that the different mixtures counteract IL-8 release with similar IC_50_, thus underlining an interaction among individual components occurring in the mixture. However, negative interactions were clearly excluded. 

**Figure 7:**
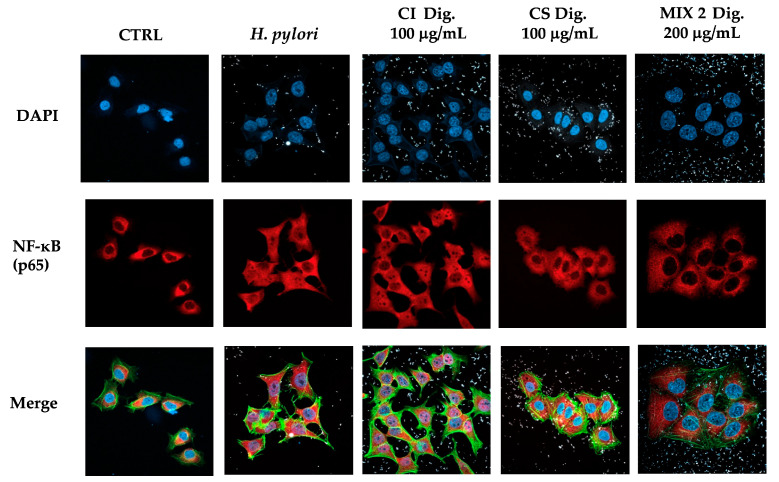
Effect of a combinations (Mix 2 Dig., 50:50) of *Cistus* × *incanus* L. (CI Dig.) and *Castanea sativa* Mill. (CS Dig.) extracts on the nuclear translocation of NF-κB (p65) induced by *H. pylori* in GES-1 cells. GES-1 cells were infected with *H. pylori* (50:1) and treated with Mix 2 Dig for 1 h. NF-κB (subunit p65, red) translocation into nuclei (blue) was measured by immunofluorescence (63× objective, 50 μm). Merged images include β-actin (green) and bacterial staining (CFSE, white).

According to results on the three mixtures, we selected Mix 2 (50:50) in the following experiments concerning the nuclear translocation of NF-κB (Figure 7) to verify the maintenance of the inhibitory effect. As expected, CS 100 μg/mL affected the translocation of NF-κB, while CI showed a negligible activity at the same concentration. In line with our previous findings, their combination reflected the bioactivity of CS, again excluding antagonistic mechanisms.

Finally, we verified the role of the combinations for the anti-adhesive effect against *H. pylori*. In this experimental contest, we previously observed an opposite behavior with respect to NF-κB impairment: CS showed inhibitory activity at concentrations higher than CI. In fact, CS was still unable to contrast the bacterial adhesion, in contrast to CI, at the concentration of 100 μg/mL.

**Figure 8:**
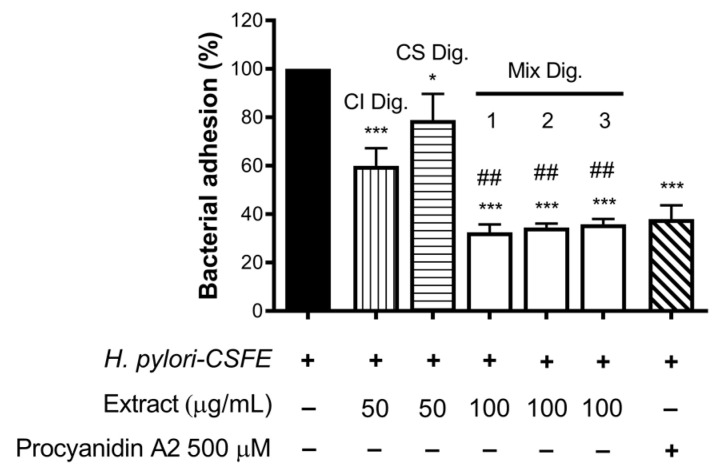
Effect of *Cistus* × *incanus* L. (CI Dig.) and *Castanea sativa* Mill. (CS Dig.) extracts on the adhesion of *H. pylori* to GES-1 cells. GES-1 cells were infected with *H. pylori–CSFE* (50:1) and treated with CI Dig. (vertical lines, 50 μg/mL), CS Dig. (horizontal lines, 50 μg/mL), or their combination (Mix, 100 μg/mL) for 1 h during infection. Procyanidin A2 (PA, 500 μM) was used as a reference inhibitor. Bacterial adhesion was measured by FACS analysis. Mix 1, combination of CI/CS Dig. 25:75; Mix 2, combination of CI/CS Dig. 50:50; Mix 3, combination of CI/CS Dig. 25:75. Results (*n* = 3) were expressed as a percentage (%) of the mean bacterial adhesion (MFI) ± SEM, relative to untreated *H. pylori* (black bar). * *p* < 0.05, *** *p* < 0.001 vs. *H. pylori;* ## *p* < 0.01 vs. CS Dig. MFI, median fluorescence intensity.

Of note, their combinations at fixed concentration (Mix 1, 2, and 3) showed a strong inhibitory effect, regardless of the ratio among the extracts (Figure 8). The anti-adhesive effect of Mix 2 (50:50), namely −66%, was slightly greater than the sum of the individual extracts (−40% and −21%, respectively, for CI Dig. And CS Dig.); however, it was not statistically different from that of CI Dig. 50 μg/mL alone. On the contrary, as expected, it was statistically different from CS Dig. 50 μg/mL. 

According with observations for IL-8, the inhibitory activity of Mix 1, 2, or 3 against *H. pylori* adhesion was mostly explained by additional effects. Again, it was interesting to exclude negative interactions and demonstrate a complementary bioactivity, including anti-inflammatory and antibacterial properties.

## 4. Discussion

Polar extracts from *Cistus* × *incanus* L. have been previously characterized for the presence of polyphenols, including flavonoids, proanthocyanidins, and hydrolysable tannins [2,3,4]. At the present state, the most investigated pharmacological activities are the antiviral and antibacterial, which have been confirmed by clinical trials on upper-respiratory-tract infections [7,11]. Despite the traditional use of *Cistus* spp. for gastrointestinal ailments, few studies suggested a potential role in gastritis treatment [5,6,23]. 

We previously showed that leaf extract from *Castanea sativa* Mill., a well-known source of hydrolysable tannins, may counteract the effect of *H. pylori* infection in human gastric cells [19]. Herein, we investigated for the first time the antibacterial and anti-inflammatory properties of a hydroalcoholic extract from *Cistus* × *incanus* L. (CI), in the same co-culture model, exploring the efficacy of a combination of both extracts previously mentioned.

TLC and colorimetric analysis (Table 2) indicated that polyphenols are highly present in the extract, among which the most representative sub-classes were vanillin-reactive compounds, such as flavan-3-ols, catechins, and oligomeric proanthocyanidins. The presence of the latter was further confirmed by n-butanol assay, in line with previously reported analysis [4]. To obtain more insights into the stability of bioactive compounds, the colorimetric tests were also performed on CI after simulated gastric digestion (CI Dig.). Vanillin-reactive compounds were partially stable after gastric simulated digestion, while flavonols were totally preserved, thus suggesting the potential maintenance of their biological activity in the gastric environment (Table 2). 

The LC–MS analysis (Table 3) confirmed the presence of several flavonol-glycosides, low amounts of monomeric and dimeric catechins, and ellagitannins, including punicalagin. However, the LC–MS analysis showed a particularly complex phytochemical profile, with many compounds but none of them predominant. This observation leads us to believe that the anti-inflammatory and antibacterial effects derive from multiple interactions among polyphenols occurring in the extract and should therefore be ascribed to the overall phytocomplex. 

Regarding the biological properties, CI lowered the IL-8 secretion by GES-1 cells infected by *H. pylori*, with IC_50_ below 32 μg/mL (Figure 1). We supposed that the inhibitory mechanism could at least in part involve the NF-κB pathway, which was affected at the concentration of 200 μg/mL (Figure 2).

Moreover, CI interfered with the adhesion of *H. pylori* to epithelial gastric cells, within the concentration range of 50–200 μg/mL. We supposed that the main mechanism was a direct impact on the bacterial viability, although indirect effects involving the host cell might have occurred (Figure 3). Accordingly, the bacterial growth was inhibited, although at higher concentrations (MIC = 250 μg/mL) (Figure 4). On the contrary, we excluded the possibility that CI could act through the reduction of biofilm formation or interfering with the urease activity (Figure 4C and Figure 5).

Our experiments showed that the decreased levels of proanthocyanidins observed after the simulation of the gastric digestion paralleled with a moderate impact on both the anti-inflammatory and antibacterial effects. On the contrary, the level of flavonols was not reduced by simulated digestion and paralleled with the conservation of the anti-adhesive effect. These observations may reflect the involvement of proanthocyanidins in the anti-inflammatory activity, along with flavonoids.

Accordingly, proanthocyanidins were considered the main fraction responsible for COX-2 inhibition and clinical improvement of respiratory-tract infections [4,7,11]. Other articles, including ours [20], attributed anti-inflammatory and anti-ulcer properties to the same polyphenolic class. Moreover, other authors also attributed an anti-adhesive effect on *H. pylori* to catechin-based proanthocyanidins from *Peumus boldus* Molina [14]. Similarly, the potential role of flavonols as antimicrobial and anti-inflammatory compounds in *H. pylori*-related gastritis was demonstrated by in vitro and in vivo studies, although anti-adhesive properties were poorly investigated (e.g., see [36,37]).

The overall data sustain the importance of proanthocyanidins and flavonoids, with specific reference to flavonols, for the biological activity of CI. However, flavonols were underlined as the main bioactive fraction stable in the gastric environment.

Of note, the polyphenolic composition and biological activity of *Cistus* × *incanus* L. and *Castanea sativa* Mill. extracts only partially overlapped [19,38]. The antibacterial effect was prevalent for the first and the anti-inflammatory for the second. Moreover, properties from *Castanea sativa* Mill. leaf extracts were at least in part due to ellagitannins, which were negligible in *Cistus* × *incanus* L.

The investigation of their combination was useful to suggest a potential complementary treatment for *H. pylori*-related gastritis. A ratio of 50:50 of the two extracts (50–100 μg/mL each) demonstrated anti-bacterial and anti-inflammatory properties in gastric cells, with a plausible advantage with respect to the use of each extract alone. In fact, an inhibitory effect on IL-8 release (Figure 6), correlated with NF-κB impairment (Figure 7), was observed. Moreover, the anti-adhesive effect against *H. pylori* was confirmed (Figure 8).

The presence of additional and complementary effects of the combination of *Cistus* × *incanus* L. and *Castanea sativa* Mill. with respect to the individual extracts may represent an advantage in terms of efficacy in the treatment of gastric ailments.

## 5. Conclusions

The present work is the first demonstration of the anti-inflammatory and antibacterial activity of an hydroalcoholic extract from aerial parts of *Cistus* × *incanus* L. against *H. pylori* infection. Based on a previous work, the combination with *Castanea sativa* Mill. leaf extracts (50:50) was suggested to sustain the anti-adhesive and anti-inflammatory properties of a novel food supplement ingredient. Despite requiring in vivo and clinical studies to clearly demonstrate the relevance and safety of our findings, the biological activity was observed at concentrations of the extract in the range of milligrams, which are plausibly achievable after oral administration in humans.

## Figures and Tables

**Table 1:** Settings related to chromatographic flow and mobile phase.

Time (min)	% A	% B	Flow (mL/min)
0	95	5	0.6
1	95	5	0.6
10	0	100	0.6
15	0	100	0.6
15.1	95	5	0.6
20	95	5	0.6

**Table 2:** Results from colorimetric assays regarding the polyphenolic fraction of CI and CI Dig.

Assay	CI	CI Dig.
	Mean ± SEM	
Folin–Ciocâlteu assay (TPC) % w. GA eq./w. d.e.	25.63 ± 1.81	15.49 ± 0.17 *^a^*
AlCl_3_ (Flavonols) % w. Quercetin eq./w. d.e.	1.35 ± 0.19	1.52 ± 0.11
Vanillin assay % w. Catechin eq./w. d.e.	24.19 ± 2.61	12.53 ± 1.97 *^b^*
n-butanol/HCl % w. Cyanidin eq./w. d.e.	2.27 ± 0.19	0.03 ± 0.13 *^a^*

*^a^*: *p* < 0.005; *^b^*: *p* < 0.05 with respect to the undigested extract (CI). *n* = 3.

**Table 3:** Results from LC–MS/MS analysis of CI.

Molecule	*m*/*z*	MS/MS	μg/mg
Catechin and/or Epicatechin	289	109	0.17
Epicatechin gallate and/or Catechin gallate	441	169	0.016
Epigallocatechin and/or Gallocatechin	305	125	0.99
Epigallocatechin gallate and/or Gallocatechin gallate	457	169	0.51
Procyanidin B1 + B3	577	189	0.1
Punicalagin A + B	1083	601	0.55
Punicalin	781	601	0.37
Ellagic acid	301	145	0.55
Chlorogenic acid	353	191	0.15
Quercetin-3-glucoside and/or Hyperoside	463	301	3.92
Kaempferol-3-glucoside	447	285	0.3

## Data Availability

Data is contained within the article.
